# Redox Regulation in Age-Related Cataracts: Roles for Glutathione, Vitamin C, and the NRF2 Signaling Pathway

**DOI:** 10.3390/nu15153375

**Published:** 2023-07-29

**Authors:** Eloy Bejarano, Jasper Weinberg, Madison Clark, Allen Taylor, Sheldon Rowan, Elizabeth A. Whitcomb

**Affiliations:** 1 JM-USDA Human Nutrition Research Center on Aging, Tufts University, Boston, MA 02111, USA; eloy.bejaranofernandez@uchceu.es (E.B.); jasper.weinberg@tufts.edu (J.W.); madisonclark054@gmail.com (M.C.); allen.taylor@tufts.edu (A.T.); sheldon.rowan@tufts.edu (S.R.); 2 School of Health Sciences and Veterinary, Universidad CEU Cardenal Herrera, CEU Universities, 46113 Valencia, Spain; 3 Friedman School of Nutrition Science and Policy, Tufts University, Boston, MA 02111, USA; 4 Department of Ophthalmology, School of Medicine, Tufts University, Boston, MA 02111, USA; 5 Department of Developmental, Chemical and Molecular Biology, Tufts University, Boston, MA 02111, USA

**Keywords:** lens, glutathione, redox, NRF2, ascorbate/vitamin C

## Abstract

Age is the biggest risk factor for cataracts, and aberrant oxidative modifications are correlated with age-related cataracts, suggesting that proper redox regulation is important for lens clarity. The lens has very high levels of antioxidants, including ascorbate and glutathione that aid in keeping the lens clear, at least in young animals and humans. We summarize current functional and genetic data supporting the hypothesis that impaired regulation of oxidative stress leads to redox dysregulation and cataract. We will focus on the essential endogenous antioxidant glutathione and the exogenous antioxidant vitamin C/ascorbate. Additionally, gene expression in response to oxidative stress is regulated in part by the transcription factor NRF2 (nuclear factor erythroid 2-related factor 2 [NFE2L2]), thus we will summarize our data regarding cataracts in *Nrf2-/-* mice. In this work, we discuss the function and integration of these capacities with the objective of maintaining lens clarity.

## 1. Introduction

A cataract is the loss of transparency of the normally clear eye lens. Opacification of the eye lens is the leading cause of blindness worldwide, representing 40% of total blindness [1]. It involves the aggregation of proteins due to the formation of many non-covalent and covalent crosslinks including non-native disulfide bonds [2]. In most cases these intermolecular bonds involved oxidative influences, thus the protein precipitation in cataracts reflects the redox status of the lens milieu. A robust antioxidant defense system plays a vital role in minimizing oxidative insult and protein aggregation to preserve lens transparency. In this review, we will focus on the regulation of the essential endogenous antioxidant glutathione, the exogenous antioxidant vitamin C/ascorbate, and the transcriptional activator NRF2 (nuclear factor erythroid 2-related factor 2 [NFE2L2]) and their roles in the development or prevention of cataract.

## 2. Lens Structure and Cataract

**Figure 1:**
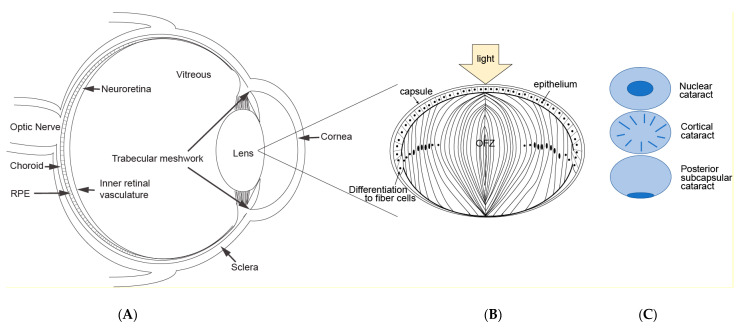
Eye and lens structure and cataract. (**A**) Cross section of the eye showing light passing through the cornea and lens. (**B**) Lens section illustrating the epithelial layer, fiber cell differentiation and the organelle-free zone. (**C**) different cataract types.

The function of the lens is to focus light onto the retina (Figure 1A). To accomplish this, the lens must remain clear. Because the lens is positioned at the front of the eye, and most lens proteins must exist and function for decades, it is susceptible to UV-light damage and oxidative stress [3]. A single layer of epithelial cells is found under the anterior surface of the capsule (Figure 1B). The epithelial cells at the germinative region divide, migrate posteriorly and differentiate into lens fibers. The lens continues to grow and differentiate throughout the lifespan of the organism, with only the epithelium proliferating. Continuous differentiation from epithelia to fiber cells results in lens growth (reviewed in [4]).

In the young lens, the flexibility of the fibers, particularly cortical, newly differentiated fiber cells, facilitates lens accommodation, allowing the eye to focus on images both near and far [5]. New cells are formed throughout life, but older cells are not lost. Instead, they are compressed and compacted into the center or nucleus of the lens [6]. The transparency of the lens is made possible by a tight configuration of lens fibers. During differentiation, there is coincident dehydration of the fibers and the fiber cells change their gene expression profiles to produce high levels of crystallin proteins which are essential for the optical properties of the lens (reviewed in [4]). Crystallin proteins contain many cysteine amino acids and thus are highly susceptible to undesired modifications during redox imbalance. Thus, redox in the lens must be tightly controlled in order for these proteins to maintain their proper structure and function.

The lens fiber cells also undergo programmed removal of their organelles, resulting in a central “organelle free zone” or OFZ (Figure 1B). While the removal of the organelles is essential for lens clarity, the lack of protein transcription and translation machinery in the OFZ results in little to no protein turnover. Thus, lens proteins are subject to cumulative oxidative insults throughout the lifespan of the organism. As the lens ages, its proteins are photo-oxidatively damaged, crosslinked, aggregate, precipitate, and accumulate in lens opacities [7]. Dysfunction of the lens due to opacification is called cataracts. The term “age-related” cataract is used to distinguish lens opacification associated with old age from opacification associated with other causes, such as congenital and metabolic disorders, or opacification induced by medication, trauma, or high-energy radiation. 

There are three main types of cataracts that are phenotypically distinguishable: nuclear, cortical, and posterior subcapsular (Figure 1C). Additionally, mixed cataracts may exhibit opacities in multiple zones. The most common cataracts are nuclear and cortical cataracts. Nuclear opacities generally affect vision more because they interfere directly with the passage of light along the visual axis. Posterior subcapsular opacities occur less frequently but are also located along the visual axis and can impair vision (reviewed in [4]).

There are many genetic mutations that lead to congenital or early-onset cataracts (reviewed in [8]). However, although the risk for age-related cataracts appears to have a genetic component [9,10,11], only a few genes have been identified that lead to earlier onset or an increased risk of age-related cataracts. Mutations in the Eph receptor A2 (*EPHA2*) lead to cortical cataracts in humans and in mouse models [12]. While mutations in the crystallin genes generally lead to congenital cataracts, mutations in αA crystallin (*CRYAA*) can also lead to an early onset age-related cataract [13]. The Emory mouse, potentially caused by a mutation in *Abhd12* [14], has an increased risk of age-related cataracts that can be ameliorated by a calorie-restricted diet [15]. Furthermore, we recently demonstrated that deletion of the antioxidant transcription factor *NRF2/NFE2L2* leads to age-related cataracts in both male and female mice [16]

Oxidative stress plays a key role in age-related cataract etiology. Excessive oxygen and its reactive metabolites have been related to an enhanced risk of cataracts. Increased cataracts have been observed in patients treated with hyperbaric oxygen therapy [17] and in guinea pigs subjected to hyperbaric oxygen treatment [18]. Smoking and tobacco confer oxidative stress and their use is associated with increased risk of cataract and nuclear sclerosis through increased oxidative stress and diminished levels of antioxidants [19,20]. In order to combat oxidative stress, the lens exploits a number of antioxidant enzymes and processes which can counterbalance the oxidative insults. A few studies have investigated the total antioxidant capacity with regard to human age-related cataracts. Kisic et al. showed that the levels of GSH, total sulfhydryl groups, ascorbate, and ferric-reducing capacity of the lens were decreased in late-stage vs. early cataracts [21]. Additionally, total antioxidant capacity and ascorbate levels in the aqueous humor were inversely related to cataract severity [22].

**Figure 2:**
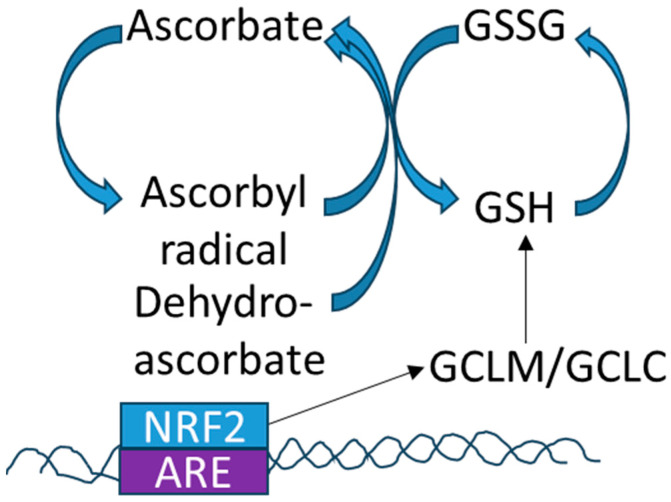
Interactions between antioxidant pathways. NRF2 increases the transcription of GCLM and GCLC, rate limiting enzymes in glutathione synthesis. GSH and ascorbate coordinate cellular redox by reducing and oxidizing each other.

This review will focus on the most abundant antioxidants in the lens: glutathione and vitamin C/ascorbate. There is coordination between ascorbate and glutathione redox regulations as each converts the other from the oxidized to the reduced form. Additionally, when activated, the transcription factor, NRF2 binds to the antioxidant response element (ARE) and directs the synthesis of the rate-limiting enzymes responsible for glutathione synthesis: glutamate–cysteine ligase (GCLC) and glutamate-cysteine ligase modifier subunit (GCLM). Thus, the glutathione, ascorbate and NRF2 pathways are interconnected to regulate cellular redox status in the lens (Figure 2).

## 3. Glutathione and the Lens

### 3.1. Glutathione Regulation in the Lens

Glutathione (γ-glutamyl-cysteinyl-glycine) is an essential antioxidant in cells and tissues, existing primarily in the reduced form (GSH). The oxidized form is GSSG, and the GSH:GSSG ratio is a robust indicator of cellular redox status. In the eye lens, a tissue highly exposed to oxidative insults, GSH is found at an extraordinarily high concentration, but the spatial distribution is not homogeneous [23,24,25]. GSH belongs to an antioxidant network that includes superoxide dismutase and catalase along with other molecules with antioxidant properties including lutein, zeaxanthin, and vitamins E and C. All these components act in concert to neutralize the damage caused by reactive oxygen species such as superoxide, hydrogen peroxide, or hydroxyl radicals. As a primary antioxidant in the lens, GSH scavenges hydroxyl radicals, participates in the biosynthesis of vitamins C and E, and is a cofactor for different key enzymes [26,27,28,29].

Homeostatic concentrations of GSH vary from the micromolar range in the extracellular milieu to the millimolar range in the intracellular environment, and decreased GSH has been found in cataracts [30,31,32]. Steady-state intracellular levels depend on intracellular GSH biosynthesis, direct uptake from the ocular humors, GSH recycling from oxidized glutathione (GSSG), export of GSH, and degradation mediated by γ-glutamyl transpeptidases [3]. A fine-tuned balance between all these pathways is key to maintaining the proper levels of GSH, preventing oxidative damage by reactive oxygen species in the lens [3].

### 3.2. Mechanisms Involved in the Maintenance of GSH Homeostasis in the Lens

One of the most striking features of the lens is that the different pathways that modulate GSH levels are distributed unevenly, and this spatial compartmentalization allows for the establishment of a GSH gradient [3]. Glutathione is a tripeptide whose biosynthesis involves three different amino acids (glutamate, cysteine, and glycine) and the sequential action of two enzymes. The first and rate-limiting enzyme in GSH synthesis is the glutamate–cysteine ligase (GCL) which forms γ-glutamylcysteine from L-glutamate and cysteine. Glutathione synthetase (GSS) then adds a glycine to the C-terminus of γ-glutamylcysteine. Intracellular GSH synthesis is active exclusively in the epithelium and cortical fibers upon the uptake of precursor amino acids from the aqueous and vitreous humors via the amino acid transporters GLYT and EAAT located in the outer cortex [33,34,35]. In addition, multiple reports suggest that uptake of GSH can occur directly from the ocular humors, although the proteins involved in this import have not been characterized, especially in the human lens [36,37,38,39,40]. A third source of GSH is the regeneration of GSH from GSSG through the action of the enzyme glutathione reductase, whose activity is high only in the cortex. In vitro studies suggest that the eye lens can export up to 20% of its total GSH in a few hours, although the transporter involved in lens GSH efflux remains to be identified [41,42]. γ-glutamyl transpeptidase is found in the lens epithelium and probably degrades exported GSH into its constituent amino acids which are released for new glutathione synthesis [43]. The contribution of this enzyme in supplying new amino acids for glutathione biosynthesis is controversial in the lens. The low levels of the enzyme in lenses compared to other ocular tissues do not seem to point toward a major role in the modulation of lenticular GSH; however, GSH levels are significantly increased in cultured lenses upon exposure to a γ-glutamyl transpeptidase inhibitor [42].

### 3.3. GSH Levels in Aging and Cataract

Age is the biggest risk factor for cataract, and the accumulation of oxidatively modified proteins are a hallmark of age-related nuclear cataract. GSH levels are differentially affected by advancing age, and significant decreases in the GSH:GSSG ratio are found in cataractous lenses compared to normal lenses [26,44,45,46]. Of note, a decrease in cataractous tissue is not observed for all antioxidant molecules; for example, no differences are found for the carotenoids lutein-zeaxanthin between normal lenses and cataractous lenses [47].

The depletion of GSH with advancing age makes the ocular tissues more susceptible to permanent tissue damage, ultimately compromising lens transparency. There is a general agreement that age-related GSH depletion is a major factor in the development of age-related nuclear cataracts (reviewed in [3]). The depletion of GSH is not uniform, taking place mostly in the center of the lens, and different pathways involved in the maintenance of GSH are affected with age (reviewed in [3]). The age-related decline of enzymes involved in GSH biosynthesis has been proposed as an Achilles’ heel in the homeostasis of lens GSH [48]. However, other activities are also impacted by advancing age. For example, decreased glutathione reductase activity was found in cataractous lenses, suggesting that a compromised regeneration system might reduce the rate of GSH recycling [49]. Also, the reduced activity of γ-glutamyl transpeptidase was found in cataractous lenses, suggesting that compromised recycling of amino acids to participate in intracellular GSH biosynthesis could be behind the pathology [50].

It is thought that the flux of GSH from the cortex to the center of the lens may occur using two different mechanisms: diffusion of cortical GSH through intercellular channels via gap junctions [51] and delivery of extracellular GSH using the internal microcirculation system plus the action of GSH transporters in the center of the lens [52]. Deficient transport of GSH from the cortex to the nucleus might participate in the regional depletion of GSH seen in cataractous lenses if the microcirculation system and/or the gap junctional intercellular communication is compromised with age.

### 3.4. In Vivo Analysis of GSH Deficiency and Cataracts

The use of different animal models with dysregulated GSH metabolism has emerged as a valuable approach to getting a better understanding of the intimate relationship among GSH, redox status, and cataracts. These in vivo models utilize genetic and pharmacological approaches in rodents to dysregulate GSH.

Pharmacological agents can be used to induce GSH deficit. For example, diethyl maleate is a GSH-depleting agent shown to enhance cataractogenicity [53]. Diethyl maleate conjugates with GSH and these conjugates are expelled, lowering intracellular GSH. However, diethyl maleate is not highly specific for GSH, thus it is not recommended for the long-term depletion of GSH. Selective inhibitors of the enzyme GCL are much more efficient tools for GSH depletion. Buthionine sulfoximine is a specific γ-glutamyl cysteine synthetase inhibitor that lowers total and reduced glutathione, increases malondialdehyde, and induces cataracts [54,55,56,57]. In postnatal day eight mice, buthionine sulfoximine induces a rapid deterioration of lens fibers and opacity is developed within 2–3 days after injection which correlates with altered protein biosynthesis [54,55]. Compounds with free radical scavenger properties such N-acetylcysteine amide, acetyl-L-carnitine, or ascorbate prevent buthionine sulfoximine-induced cataract formation in Wistar rats. Recently, the dietary intake of taurine was also shown to be able to protect against GSH depletion in the rabbit lens, suggesting that taurine also acts as an antioxidant in the lens [54,58,59,60,61].

Animal models targeting GSH biogenesis genetically are also valuable tools to investigate GSH depletion in the context of lens opacity. GSH is the major thiol antioxidant and the disruption of GSH synthesis in the whole organism is lethal at an early embryonic stage [62,63]. Hepatic-specific glutamate–cysteine ligase knockout mice develop steatosis, mitochondrial injury, and liver failure. These mice deficient for hepatic GSH synthesis die at approximately one month of age [64], highlighting the importance of GSH as a biological determinant of tissue homeostasis. Cre-lox technology has allowed researchers to develop lens-specific knockouts for GCLC where de novo GSH synthesis is completely abolished, lowering levels of intralenticular GSH. These mice, homozygous lens GSH knockout mice (LEGSKO), start to develop nuclear cataracts at four months [65]. Transcriptomics and proteomics analysis revealed that the lens adapts to the GSH-deficiency by modulating detoxifying genes, EMT signaling, transport systems, and lipid homeostasis. Despite these compensatory mechanisms, the LEGSKO mice develop severe nuclear cataracts [61,66]. Mice lacking *Nrf2*, a key transcriptional modulator of GCLC, start developing opacities at nine months and develop various age-related cataracts including advanced cortical, posterior subcapsular, anterior subcapsular, and nuclear cataracts (see below) [16]. In a buthionine sulfoximine treatment model, the addition of γ-glutamylcysteine ethyl ester significantly increased glutathione in the lens [67]. Furthermore, oral administration of γ-glutamylcysteine has been shown to increase the levels of glutathione in human trials [68]. Thus, supplementation of γ-glutamylcysteine may be an indirect way to increase glutathione levels in the lens. Additionally, supplementation of taurine has been shown to increase glutathione in the lens and ameliorate cataracts in animal models [60]. Thus, these compounds may be useful in treating human age-related cataracts.

### 3.5. Glutathione and Protein Homeostasis

The balance between reduced and oxidized glutathione can affect protein quality control in the lens. A major regulator of protein quality control is the ubiquitin pathway. Ubiquitination regulates nearly every pathway in the cell by regulating stability, intracellular trafficking, and function of proteins. The ubiquitin pathway is essential in controlling proteostasis; the balance between protein production and destruction that is essential for cellular homeostasis [69]. There are three main types of ubiquitination enzymes, E1s, ubiquitin-activating enzymes, E2s, ubiquitin-conjugating enzymes, and E3s, ubiquitin ligases. The E1s, E2s, and many of the E3 enzymes are thiol-reactive, and thus their activities are affected by changes in cellular redox. High levels of GSSG caused by oxidative stress can decrease the charging of ubiquitin E1 and E2 proteins. This results in diminished ubiquitination upon oxidative stress in both the lens and retina [70,71]. Additionally, treatment of the normally stable lens structural protein, γC crystallin with glutathione leads to its unfolding and this is associated with more rapid degradation [72]. Thus, the ratio of GSH:GSSG affects the activity of the enzymes that target proteins for destruction as well as altering the stability of substrates, indicating a role for glutathione in maintaining proteostasis. Thus, changes in GSH:GSSG observed during aging and cataracts could be contributing to disrupted proteostasis in the lens.

## 4. Vitamin C and the Lens

### 4.1. Redox and Vitamin C

Endogenous and exogenous antioxidants interact to contribute to the overall redox status in the lens. Ascorbate or vitamin C is probably the most effective and least toxic antioxidant identified in mammals. It can scavenge free radicals such as superoxide, hydrogen peroxide, and singlet oxygen [73,74]. Ascorbate interacts with the glutathione cycle, the thioredoxin cycle and the tocopherol/vitamin E cycle to control redox in the cell (Figure 2 reviewed in [4]). There is a positive relationship between glutathione and vitamin C/ascorbate. Ascorbate can increase GSH directly and by affecting the activities of GSH-peroxidase and GSSG-reductase (reviewed in [75]). In a prospective study of 200 people, those with adequate levels of ascorbate had higher levels of plasma GSH than those with lower levels [76]. The lower levels of ascorbate and GSH were also correlated with increases in oxidative stress markers in the plasma. Supplementation of ascorbate increased GSH levels in lymphocytes [77] and red blood cells [78] in intervention trials.

In the lens, ascorbate can reduce GSSG to GSH and GSH can reduce dehydroascorbate to ascorbate (see Figure 2). Thus, there is coordination between ascorbate and glutathione redox regulation. Glutathione improves the antioxidant capacity of ascorbate and can ameliorate the toxicity of high levels of ascorbate on lens cells in culture [79]. Depletion of GSH from lens epithelial cells can increase their susceptibility to oxidative stress. This can be ameliorated by the addition of ascorbate or tocopherol/vitamin E [80].

### 4.2. Vitamin C Levels in Lens and Cataract

Vitamin C is not synthesized in humans and must be taken in through dietary consumption. Epidemiologic studies suggest that insufficiency of ascorbate increases the risk of cataracts (reviewed in [4]). While the recommended daily intake of vitamin C is 75–90 mg/day, intake of 135 mg/day or blood levels of at least 49 mM are correlated with decreased risk of cortical, nuclear, and posterior subcapsular cataracts [81]. However, the impact of vitamin C is not equal for all types of cataracts. Here, we summarize the most relevant literature for each type of opacity. See Table 1 for a summary of studies.

Cortical Cataract: Several studies indicate a protective role for vitamin C in reducing the risk of cortical cataracts. The cross-sectional INDEYE study of rural Indians indicated that people with the highest plasma levels of vitamin C had a 35% reduced risk of cortical cataracts compared with those with the lowest levels [82]. This effect appeared to be driven by participants living in the southern rather than the northern part of India [83]. This geographic difference is of interest because there is a “cataract belt” of high cataract prevalence in certain regions of India. The benefits of vitamin C are supported by the prospective analysis in the Nutrition Vision Project (NVP), a subset of the Nurses’ Health Study. This study showed that, among women aged ≤ 60 years, consumption of at least 363 mg/day of vitamin C was associated with a 57% decrease in risk of developing cortical cataracts compared with women who consumed less than 140 mg/day. Additionally, women who took supplemental vitamin C for at least 10 years had significantly fewer cortical lens opacities than those who did not take supplements [84].

Nuclear cataract: Vitamin C intake is also correlated with a reduced risk of nuclear cataracts. Approximately a 40% decreased risk has been reported for people with intakes above 135 mg/day or blood concentrations of 6 μM (reviewed in [4]). Long-term elevated intake or use of supplements was also associated with a decreased risk of nuclear cataracts. In the INDEYE study, those with plasma levels of vitamin C in the highest tertile had decreased risk for nuclear cataracts compared with the lowest tertile [82,83]. Notably, this benefit was observed in participants from both northern and southern India.

Posterior subcapsular cataract: Retrospective studies suggest that elevating intake and plasma levels of vitamin C may confer weak protection for posterior subcapsular cataracts among those with an intake of at least 491 mg/day or blood levels above 49 μM [81]. A similar decrease was also observed in the INDEYE study in both the northern and southern study centers [82,83].

Intervention trials to increase vitamin C intake as a means to ameliorate cataracts have been inconclusive. Many of the intervention trials include multiple antioxidants in the supplements making it challenging to come to any conclusions regarding vitamin C specifically. The Roche European American Cataract Trial (REACT) had participants taking supplements with beta carotene, vitamin C, and vitamin E for three years. This trial, although with a small sample size (158 participants total), did show modest, statistically significant decreases in cataracts in both the US and the UK [85]. The Antioxidants in Prevention of Cataract study (APC), a five-year randomized controlled trial of nearly 800 patients with mild cataracts in southern India also supplemented individuals with beta carotene, vitamin C and vitamin E. Despite high compliance with the study subjects, there were no differences between groups [86]. The Physicians Health Study, with over 11,000 male participants, supplemented with a daily multivitamin, alternate day vitamin E and vitamin C, showed positive effects with regards to cataract incidence. Overall, there was a significant 9% lower risk of all cataracts in the supplement group compared to the placebo. There was also a small, but non-significant reduction in cortical cataracts. They observed no differences in posterior subcapsular cataracts or cataract extraction [87]. A recent meta-analysis of randomized controlled trials showed that vitamin C supplementation decreases the risk of age-related cataracts [88]. Thus, while vitamin C intake correlates with decreased risk of cataracts, there is no consensus as to whether supplementation is effective. In fact, high levels of supplementation may be detrimental (see below).

### 4.3. Too Much of a Good Thing?

Although increased intake of vitamin C is correlated with protection from cataracts, there is also evidence that too much can be detrimental. Fan et al. developed a transgenic mouse overexpressing hSVCT2, a sodium-dependent vitamin C transporter [89,90]. These mice have increased levels of vitamin C in their lenses, especially the oxidized form, dehydorascorbate, and develop cataracts by 12 months of age. Furthermore, the hSVCT2 lenses have increased levels of advanced glycation end products (AGEs) upon aging [89]. This is relevant because ascorbic acid glycation contributes to cataractogenesis and ascorbate-derived AGEs are increased upon aging in human cataracts [91]. While the majority of epidemiologic studies show a protective role for vitamin C and cataracts, there are several studies that indicate an overabundance of vitamin C may contribute to increases in cataracts. In a Swedish cohort of women taking high doses of supplemental vitamin C (~1000 mg/day), a 38% increased risk of cataracts was observed [92]. Another study showed a 36% increased risk of cataracts in men supplemented with high doses of vitamin C alone (~1000 mg/day), but not with men taking vitamin C in conjunction with a multivitamin [93]. These studies suggest that supplementation of high levels of vitamin C on its own may be deleterious but as part of a varied diet or a multivitamin, vitamin C may be beneficial for reducing cataract risk.

**Table 1:** Human studies relating Vitamin C and age-related cataracts.

Association	Type of Study	Findings	Ref.
INDEYE	Cross-sectional	Decreased risk of all catraract types in highest vs. lowest tertiles of plasma vitamin C	[82,83]
Nutrition and Vision Project	Retrospective	Decreased risk of cataract with ≥363 mg/day Vitamin C	[83]
Intervention	Length of Intervention	Findings	Ref.
REACT	3 years	Small decrease in opacification upon supplementation	[85]
APC	5 years	No difference in cataract upon supplememtation	[86]
PHS	11.2 years	9% lower risk in multivitamin supplemented group	[87]
Swedish mammography cohort	8.2 years	25% increased risk with high dose supplementation of vitamin C	[92]
Cohort of Swedish men	8.4 years	21% increased risk with high dose supplementation of vitamin C alone	[93]

## 5. NRF2 and Cataract

### 5.1. NRF2 Antioxidant Response

The NRF2 (nuclear factor erythroid 2-related factor 2 [NFE2L2]) transcription factor acts as a primary cellular defense mechanism against oxidative stress. NRF2 is normally located in the cytoplasm, bound to the cullin-3 (CUL3) and the adaptor protein Kelch-like ECH-associated protein 1 (KEAP1). Under stress-free conditions, NRF2 is ubiquitinated by the CUL3 complex, and targeted for degradation by the proteasome [94,95]. Upon oxidative stress, NRF2 is released from KEAP1, translocates to the nucleus and activates the transcription of a set of antioxidant response element (ARE, see Figure 2) genes to facilitate a rapid response to oxidative stress [94,96].

Genes activated by NRF2 are largely cytoprotective, involving pathways including glutathione synthesis, as well as detoxification and elimination of reactive oxygen species [94]. Thus, it is not surprising that the inhibition of NRF2 subsequently decreases the capacity for the cell to respond to oxidative stress, which is exacerbated with increasing age [94,97]. NRF2 directly activates transcription of the rate-limiting enzymes responsible for glutathione synthesis, GCLC, and GCLM (glutamate-cysteine ligase modifier subunit, see Figure 2), and regulates glutathione regeneration [98]. Activation of the NRF2 pathway is important in the protection against oxidative stress in many disease states including fatty liver disease [99], pre-eclampsia [100], pulmonary disease [101], cardiovascular disease, and neurodegenerative disease (reviewed in [102]). In contrast, hyperactivion of the NRF2 pathway may support the survival of cancer cells [103]. The association between reduced downstream antioxidant effects due to decreased NRF2 activity, evident with age and in age-related cataracts, has garnered attention for NRF2 as a therapeutic target for cataract treatment and prevention [96].

### 5.2. NRF2 in the Lens

There is growing literature about the NRF2-GSH axis in lens functions. Human lens epithelial cells (HLECs), broadly used as a surrogate for whole lens biology, when treated with suppressors of NRF2 activity recapitulate markers of protein aggregation as expected for cataractogenesis [104,105,106,107,108,109]. Conversely, treatment with antioxidant compounds, including those that induce NRF2 transcription and activation, protects against selenite-induced cataracts [110,111]. Similarly, activators of NRF2 can protect HLECs from features of cataracts that are induced under oxidative stress due to high glucose, hydrogen peroxide, or homocysteine treatments [107,108,112,113,114]. Analysis of age-related cataractous lenses from human diabetic or non-diabetic samples showed decreased methylation of the KEAP1 promoter, which in turn upregulates KEAP1 expression, and inhibits the NRF2 antioxidant response [111,115].

**Figure 3:**
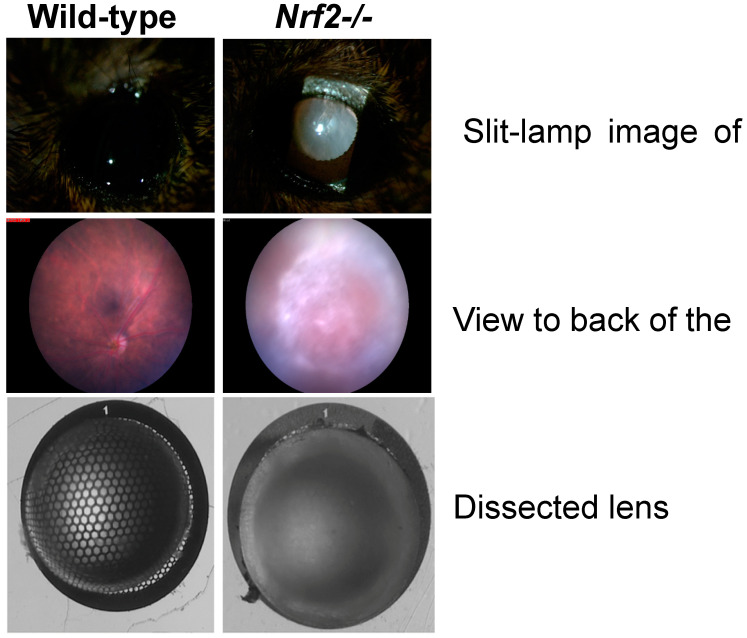
Deletion of *Nrf2* leads to age-related cataracts. Examples are shown of 18 months wild-type or *Nrf2-/-* lenses. These cataracts contain nuclear, cortical and capsular opacities, resulting in the blocking of light to the retina.

Downstream targets of NRF2, including glutathione synthesis, thioredoxin reductase, glutathione reductase, thioltransferases, and levels of all these antioxidant enzymatic functions have been shown to be decreased in cataracts [28,32,116,117,118]. We recently demonstrated that male and female NRF2 knockout mice (*Nrf2-/-*) have increased frequency and severity of cataracts. Intriguingly, aged *Nrf2-/-* mice developed advanced cortical, posterior subcapsular, anterior subcapsular, and nuclear cataracts rather than being restricted to one type of cataract [16]. Figure 3 shows representative lenses from *Nrf2-/-* mice with the most severe (grade 4) cataract.

We previously demonstrated that low glycemic index diets protect mice from age-related macular degeneration-like phenotypes [119,120,121,122]. Additionally, epidemiologic studies indicate that people with high serum glucose have an increased risk of cataracts (reviewed in [4]). Incidence of cataracts in the *Nrf2-/-* mice was not influenced by a low or high glycemic index diet. However, we observed a modest but not statistically significant decrease in the cataract incidence in aged *Nrf2-/-* mice upon calorie restriction (CR) [16]. Similarly, cataracts in the Emory mouse are ameliorated by CR [123,124,125,126]. An increase in glutathione was also observed in the CR Emory mice, suggesting that CR may be able to compensate, at least in part, for decreased NRF2 signaling [125,127]. Others have shown that CR can compensate for some deficits associated with NRF2 deletion but not others [128]. Activation of the NRF2 pathway by nutritional means using plant-derived compounds such as sulforophane and dimethyl fumarate. oleanolic acid, curcumin, and resveratrol may be of interest for the treatment of the number of diseases (reviewed in [129,130]), it will be of interest to determine whether these foods and nutritional compounds can delay age-related cataracts.

## 6. Concluding Remarks and Future Perspectives

The role of altered redox in the etiology of cataracts is well known. A better understanding of redox regulation in cataractogenesis is imperative in order to design effective therapeutic strategies. The essential antioxidant glutathione plays an important role in controlling redox in the lens as mutations in the pathway and dysregulation in aging are correlated with cataracts. Additionally, the exogenous antioxidant ascorbate/vitamin C plays a role in regulating the redox milieu of the lens, with studies indicating that diets rich in vitamin C are protective against many types of age-related cataracts, although intervention trials have not been successful in preventing cataract and some studies suggest there may be deleterious effects of vitamin C supplementation on its own. Furthermore, the activity of the transcription factor NRF2 which stimulates a set of antioxidant response genes is essential for maintaining lens clarity in aging. Further studies into the role of diet, including CR and nutritional compounds that activate NRF2, and redox regulation in the lens may identify new targets for intervention in age-related cataracts.

## Data Availability

Data sharing not applicable.
